# Factors associated with air leak prolongation observed after lung cancer surgery

**DOI:** 10.1186/s13019-026-04355-8

**Published:** 2026-06-28

**Authors:** Satoshi Kamata, Itaru Ishida, Yuyo Suzuki, Hirotoshi Suzuki, Hiroyuki Oura

**Affiliations:** Department of Thoracic Surgery, Iwate Prefectural Central Hospital, Ueda 1-4-1, Morioka, 020-0066 Japan

**Keywords:** Air leak, Prolonged air leak, Risk factor, Score

## Abstract

**Purpose:**

Risk factors that classify patients with postoperative air leaks into prolonged-air-leak (PAL, ≥ 5 days) and non-PAL populations remain unknown. Our objectives were to identify these risk factors and devise a scoring system to predict whether air leaks observed on postoperative day 1 will progress to PAL.

**Methods:**

We conducted a retrospective examination of 329 patients after lung surgery.

**Results:**

Significant factors associated with air leaks included male sex (*p* = 0.0097), low body mass index (BMI) (*p* = 0.0016), smoking (*p* = 0.0076), pack-years (0.0017), predicted vital capacity (*p* = 0.0053), predicted forced expiratory volume in one second (*p* = 0.0352), and operative duration (*p* = 0.0118). Furthermore, in patients with air leaks on the day after surgery, significant factors associated with PAL included low BMI (*p* = 0.0248), current smoking (*p* = 0.0054), pack-years (*p* = 0.005), and significant blood loss (*p* = 0.0147). Upon classifying the patients on day 1, one point each was allotted for BMI < 23.7, pack-years ≥ 28.5, current smoker, and blood loss ≥ 38 ml. Depending upon the risk factors present, the rates of PAL observed were 0%, 7.69%, 33.33%, 70.59%, and 83.33% associated with 0, 1, 2, 3, and 4 points, respectively (*p* < 0.001).

**Conclusions:**

For the first time, we identified risk factors for the onset of postoperative air leakage and PAL.

## Introduction

Air leakage is the most frequent complication seen after lung surgery [1]; if prolonged, such leakage can lead to serious complications such as empyema. Furthermore, air leaks prolong the duration of drain placement as well as the hospital stay, which in terms of increased medical cost, further burdens the patient [2, 3]. In research using patients who had undergone surgery (including all patients who did and did not have air leaks after surgery) as the sample population, various risk factors for prolonged air leak (PAL) after surgery have been identified, with the main factors being the presence of accretion and items related to COPD [4]. Therefore, we can make a preoperative prediction of whether a patient is prone to PAL. However, there has been hardly any research on factors that separate patient populations with air leaks after surgery into patient populations with air leaks that fall into prolonged and nonprolonged populations. Perhaps the factors that increase the risk of postoperative air leakage are different from those that increase the risk of PAL. Our objectives were to investigate the risk factors for postoperative air leakage and PAL and to devise a simple score to predict whether air leakage observed on postoperative day 1 would be prolonged.

## Methods

### Patients

We conducted a retrospective cross-sectional study of 329 patients who underwent lobectomy or segmentectomy at the Department of Thoracic Surgery, Iwate Prefectural Central Hospital, between 2015 and 2019. Patients who underwent pneumonectomies or complicated ablation of the thoracic wall or diaphragm and patients who used a ventilator after surgery were excluded. The surgical care team checked for the presence or absence of air leaks daily. PAL was defined as persistent air leakage for 5 days or more. Smokers included current smokers and past smokers. Nonsmokers were defined as individuals who had never smoked. The study was approved by the Ethics Committee of Iwate Prefectural Central Hospital (October 1, 2020; number: 1078), which waived the requirement for individual patient consent because only routine patient data were used for this retrospective analysis. All experiments were performed in accordance with relevant guidelines and regulations.

### Surgical procedure

Surgery was performed under general anesthesia using differential lung ventilation. All surgical patients underwent thoracoscopy-assisted surgery with a wound size of < 8 cm. For lung resections such as lobectomy, staplers were used (Auto Suture: Covidien, Minneapolis, MN, USA). Receiver operating characteristic (ROC) curve analysis was performed to determine optimal cutoff values for continuous variables associated with PAL. Cutoff values were selected based on the Youden index. Due to the limited number of PAL events, multivariable analysis was not performed to avoid model overfitting.

### Postoperative management

We placed a 20 Fr. chest tube towards the lung apex, and a 24 Fr. chest tube towards the lung base. Using a drainage bag (Sumitomo Bakelite Co., Tokyo, Japan), continuous suction was performed at −10 cm H_2_O. When no postoperative air leak was observed, both tubes were removed on postoperative day 2. When an air leak persisted on the second day, only the tube towards the lung base was removed, while the tube towards the lung apex was removed once it had been confirmed that the air leak had resolved.

### Statistical analysis

Statistical data were analyzed using JMP^®^ 15 (SAS Institute Inc., Cary, NC, USA). When performing group comparisons, Student’s t-test was used when variables were discrete and continuous. Furthermore, a chi-square test was used for categorical variables. A p-value of < 0.05 was considered statistically significant.

## Results

### Patient classification

**Fig. 1:**
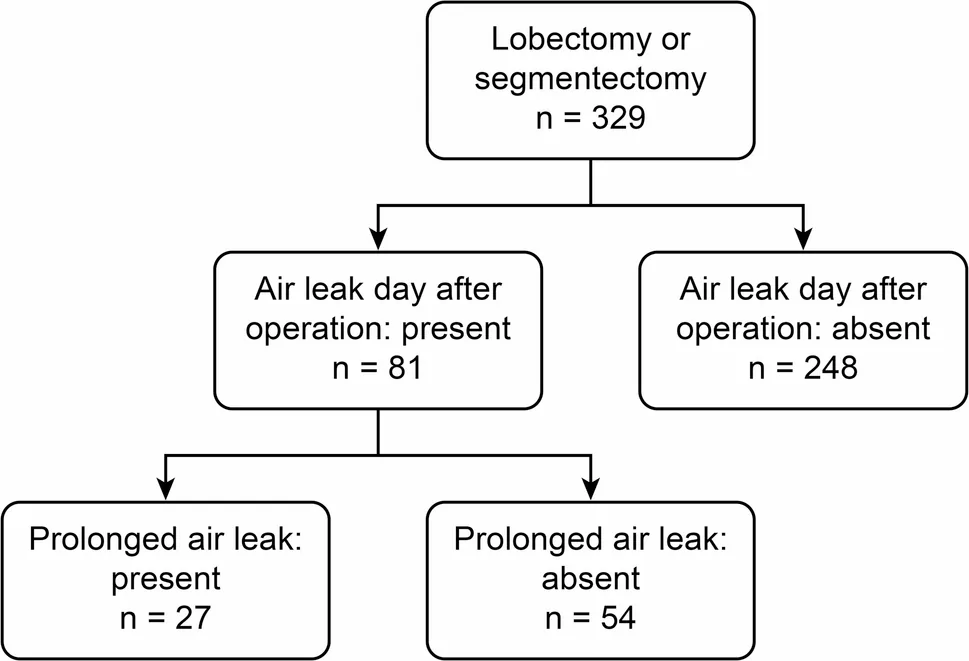
Patients classification according to air leak occurrence and prolonged air leak development

Among 329 patients who underwent lobectomy or segmentectomy, air leakage was observed on the following day in 81 patients. In 248 patients, no air leak was observed. Of the 81 patients who had air leakage the day after surgery, 27 experienced PAL. In 54 patients, PAL did not develop, and the drain was removed (Fig. 1).

### Comparison between the group with air leakage and the group without air leakage on postoperative day 1

A comparison of the patient background between groups is shown in Table 1. In the group with air leakage observed the day after surgery, there were significantly more men (*p* = 0.0097). Furthermore, the body mass index (BMI) was significantly lower (*p* = 0.0016), the proportion of smokers was significantly higher (*p* = 0.0076), and the number of pack-years was also higher (0.0017) in the group with air leakage the day after surgery. The predicted forced vital capacity % (FVC%) and predicted forced expiratory volume in one second (FEV 1.0%) were both significantly lower in the group with air leakage observed the day after surgery (*p* = 0.0053, *p* = 0.0352). We also found that the operative duration was significantly longer in the group with air leakage observed the day after surgery (*p* = 0.0118).

**Table 1:** Patient-characteristic distribution depending on the onset of air leakage at day 1 after surgery

Variable	Air leak at day 1 after surgery			
	Total	Yes	No	*p*-value
Number (%)	329	81 (25)	248 (75)	
Age, years	68.3 ± 8.8	68.9 ± 8.0	68.1 ± 9.0	0.5049
Sex, male/female	158/171	49/32	109/139	0.0097
BMI, kg/m²	23.4 ± 3.4	22.4 ± 3.4	23.7 ± 3.4	0.0016
Smoking history, no/yes	156/173	28/53	128/120	0.0076
Smoking status, current/ex/non	57/116/156	17/36/28	40/80/128	0.0281
Pack-years	20.0 ± 26.0	26.7 ± 25.5	17.9 ± 25.8	0.0017
FVC, % predicted	104.1 ± 17.2	99.7 ± 18.2	105.5 ± 16.6	0.0053
FEV₁, % predicted	101.4 ± 20.0	96.7 ± 22.5	102.9 ± 19.0	0.0352
FEV₁/FVC, %	77.1 ± 8.6	75.7 ± 13.1	77.2 ± 8.1	0.4127
Resected side, right/left	196/133	55/26	141/107	0.0786
Resected site, upper/middle/lower	173/29/127	50/6/25	123/23/102	0.1639
Operation time, minutes	197 ± 56	216 ± 72	191 ± 49	0.0118
Intraoperative blood loss, g	56 ± 79	72 ± 108	51 ± 66	0.6004

### Comparison between the group with PAL and the group without PAL among those with air leakage observed the day after surgery

Among the group with air leakage observed the day after surgery, a comparison between patients with PAL and patients without PAL is presented in Table 2. In the group that experienced PAL, the BMI was significantly lower (*p* = 0.0248), and the proportion of current smokers was significantly higher (*p* = 0.0054). In addition, the number of pack-years was significantly higher (*p* = 0.005), and the amount of blood loss was significantly greater in the PAL group (*p* = 0.0147).

**Table 2:** Patient-characteristic distribution depending on the prolongation of air leakage after postoperative day 1

Variable	Prolonged air leak			
	Total	Yes	No	*p*-value
Number (%)	81	27 (33)	54 (67)	
Age, years	68.9 ± 8.0	69.7 ± 6.2	68.4 ± 8.8	0.7331
Sex, male/female	49/32	20/7	29/25	0.0771
BMI, kg/m²	22.4 ± 3.4	21.1 ± 2.5	23.0 ± 3.7	0.0248
Smoking history, no/yes	28/53	5/22	23/31	0.0317
Smoking status, current/ex/non	17/36/28	11/11/5	6/25/23	0.0054
Pack-years	26.7 ± 25.5	38.2 ± 26.3	20.9 ± 23.2	0.005
FVC, % predicted	99.7 ± 18.2	100.0 ± 21.5	99.6 ± 16.7	0.8332
FEV₁, % predicted	96.7 ± 22.5	95.4 ± 22.8	97.3 ± 22.5	0.4689
FEV₁/FVC, %	75.7 ± 13.1	75.7 ± 13.2	77.6 ± 10.1	0.3605
Resected side, right/left	55/26	20/7	35/19	0.4001
Resected site, upper/middle/lower	50/6/25	18/2/7	32/4/18	0.7866
Operation time, minutes	216 ± 72	225 ± 83	211 ± 66	0.5444
Intraoperative blood loss, g	72 ± 108	111 ± 149	53 ± 74	0.0147

### Scores to predict PAL

**Fig. 2:**
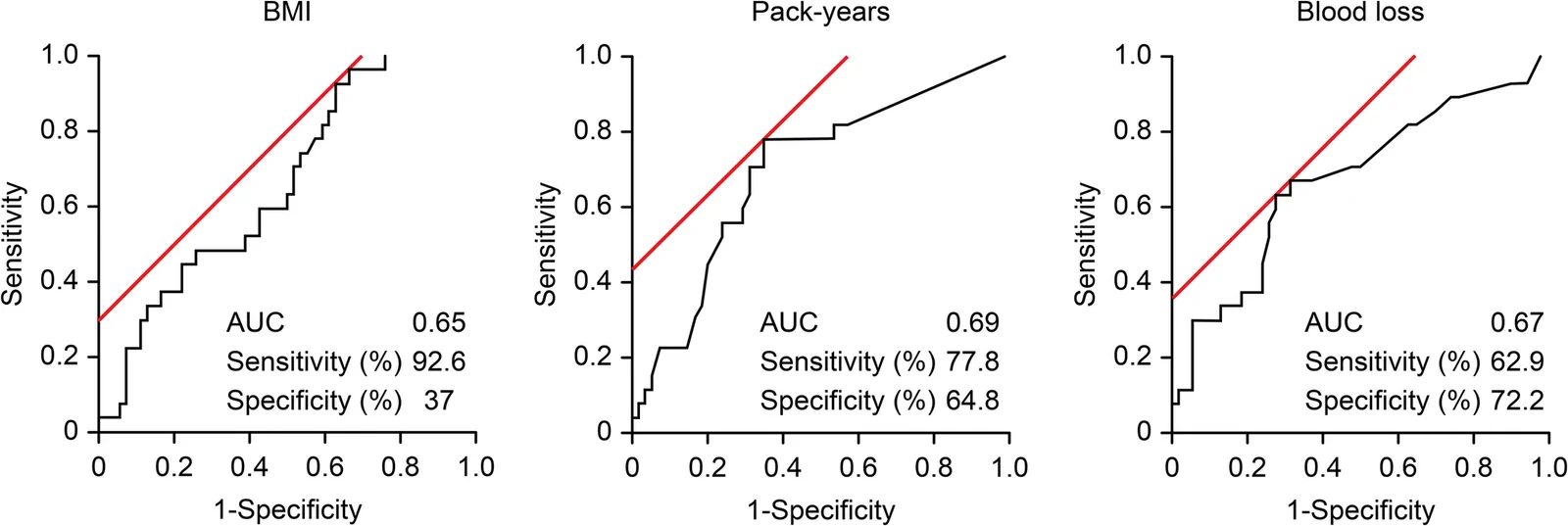
Receiver operator characteristic curve for body mass index, pack-years, and amount of blood loss in predicting risk of prolonged air leak. AUC: area under curve

In the group with air leakage observed the day after surgery, we created a receiver operator characteristic (ROC) curve (Fig. 2) and set the cutoff values for BMI, pack-years, presence or absence of current smoking habits, and amount of blood loss, which demonstrated a significant difference between the group with PAL and the group without PAL. Scoring was performed by adding one point for each of the following: BMI < 23.7, pack-years ≥ 28.5, current smoker, and blood loss ≥ 38 ml (Table 3).

**Table 3:** Prolonged-air-leak score

Question	Answer = yes
Is the patient’s BMI ≤ 23.7?	1 point
Is the patient’s pack-year ≥ 28.5?	1 point
Is the patient a current smoker?	1 point
Is the patient’s intraoperative blood loss ≥ 38 ml?	1 point

### Prediction of PAL using scores

The proportion of PAL for each potential score in the group with air leakage on postoperative day 1 is presented in Table 4. PAL was experienced by 0%, 7.69%, 33.33%, 70.59%, and 83.33% of patients in the groups with 0, 1, 2, 3, and 4 points, respectively (*p* < 0.001).

**Table 4:** PAL scoring system

PAL score	PAL			
	Number (%)	Yes	No	Frequency of PAL (%)
	81	27	54	
0	8 (9.9)	0 (0)	8 (14.8)	0
1	26 (32.1)	2 (7.4)	24 (44.4)	7.69
2	24 (29.6)	8 (29.6)	16 (29.6))	33.33
3	17 (21.0)	12 (44.5)	5 (9.3)	70.59
4	6 (7.4)	5 (18.5)	1 (1.9)	83.33

## Discussion

In the present study, we aimed to elucidate factors associated with the prolongation of air leaks observed postoperatively and to create a score to predict PAL. We identified risk factors for postoperative air leakage and PAL and created a scoring system that predicted PAL based on BMI < 23.7, pack-years ≥ 28.5, current smoking status, and blood loss ≥ 38 ml.

Previous studies examined factors related to and created scores to predict postoperative PAL in patients who underwent surgery [4]. In general, risk factors for PAL include advanced age [5], male sex [6], smoking [7, 8], low BMI [6, 7, 9], low FEV1 [2, 5, 9, 10], low albumin levels [11], upper lobectomy [10], right lobectomy [12], and adhesions [5, 6, 13]. However, few studies have investigated the presence of PAL in a population with air leaks observed the day after surgery. We therefore examined factors that predict the prolongation of air leaks observed after surgery.

In the present study, factors associated with air leak susceptibility after surgery included male sex, low BMI, history of smoking, large smoking quantity, low vital capacity, low FEV1, and long operative duration. On the other hand, among the group with an air leak observed postoperatively, risk factors for PAL included low BMI, history of smoking, being a current smoker, large smoking quantity, and extensive blood loss.

We found that BMI and smoking were associated with air leak onset as well as air leak prolongation. On the other hand, while respiratory function was associated with air leak onset, it was not associated with air leak prolongation. Current smoking and extensive blood loss were factors that were not associated with air leak onset but were in fact associated with air leak prolongation. As expected, COPD-related factors were associated with postoperative air leakage as well as air leakage prolongation. Current smoking and extensive blood loss are thought to have an adverse effect on postoperative healing and thus are associated with air leak prolongation. Factors associated with postoperative PAL include lung damage such as emphysema, low BMI, and reduced immunity related to smoking [5, 7, 14–17].

Therapeutic interventions for postoperative air leakage include repeat surgery and pleurodesis, which are considered for each patient. However, the importance and appropriate timing of therapeutic intervention are unclear, and at present, such interventions are performed on the basis of general practical experience. In the present study, we created a simple score to predict PAL. The PAL score can be easily used in routine medical practice, and planning early therapeutic intervention would be beneficial for patients thought to be at high risk of PAL.

PAL poses a burden to patients in terms of pain associated with long-term drain placement and financial costs due to additional treatment and longer hospital stays. Countermeasures for postoperative air leaks can be performed pre-, intra-, and postoperatively. Preoperative countermeasures include performing surgery with time allowed for smokers to quit smoking. Intraoperative countermeasures include performing manipulations with care, including air leak repair during surgery, while postoperative countermeasures include drain management, pleurodesis, and surgical treatment.

In patients at high risk of postoperative air leakage, caution should be exercised to prevent air leaks during surgery, and for patients at high risk of PAL, early intervention for air leakage should be considered.

The limitations of this research are that it was a retrospective investigation at a single facility and that the sample size was small. However, this pilot study provided promising results, demonstrating the importance of developing large-scale multisite investigations.

Previous studies have identified factors such as fissure completeness, adhesions, and surgical technique (e.g., fissureless vs. fissure-first) as important predictors of PAL. However, these variables were not included in the present study. This was primarily because detailed and consistent records of these intraoperative findings were not available in our retrospective dataset. Therefore, their inclusion in the analysis was not feasible.

Although multiple surgeons were involved in this study, all procedures were performed under standardized institutional protocols for thoracoscopic surgery. Nevertheless, differences in surgical technique and operator experience may have influenced the results and should be considered as a limitation.

In this study, air leaks were assessed using a conventional drainage system rather than a digital system. Although digital systems provide more objective and reproducible measurements, air leak evaluation in our institution was performed by experienced staff using standardized criteria. However, some degree of interobserver variability cannot be excluded.

Due to the relatively small number of PAL events, multivariable analysis was not performed to avoid overfitting. Instead, we focused on developing a simple and clinically applicable scoring system based on variables identified in univariable analyses.

Lobectomy and segmentectomy were analyzed together in this study. Although the extent of lung resection may influence postoperative air leak duration, we did not evaluate these procedures separately because this was an exploratory study with a limited number of PAL events. Future studies with larger cohorts should assess the influence of the extent of resection on PAL among patients with postoperative air leakage.

This study has several limitations, including its retrospective design and single-center setting. In addition, external validation is required before clinical application. Prospective validation of this scoring system is warranted in future studies.

## Conclusion

We elucidated the risk factors for postoperative air leakage and PAL and created a simple score that effectively predicts postoperative PAL. We believe this score could be applied to clinical practice to set up pre-, intra-, and postoperative countermeasures for prolonged postoperative air leaks.

## Data Availability

No datasets were generated or analysed during the current study.

## Abbreviations

PAL: Prolonged air leak
COPD: Chronic obstructive pulmonary disease
FVC: Forced vital capacity
FEV: Forced expiratory volume
BMI: Body mass index
ROC: Receiver operator characteristics
AUC: Area under the curve

## References

[1] Cerfolio RJ, Bass CS, Pask AH, Katholi CR. Predictors and treatment of persistent air leaks. Ann Thorac Surg. 2002;73:1727–30. 12078760 10.1016/s0003-4975(02)03531-2

[2] Liang S, Ivanovic J, Gilbert S, Maziak DE, Shamji FM, Sundaresan RS, et al. Quantifying the incidence and impact of postoperative prolonged alveolar air leak after pulmonary resection. J Thorac Cardiovasc Surg. 2013;145:948–54. 22982031 10.1016/j.jtcvs.2012.08.044

[3] Varela G, Jiménez MF, Novoa N, Aranda JL. Estimating hospital costs attributable to prolonged air leak in pulmonary lobectomy. Eur J Cardiothorac Surg. 2005;27:329–33. 15691691 10.1016/j.ejcts.2004.11.005

[4] Shintani Y, Funaki S, Ose N, Kanou T, Fukui E, Minami M. Preoperative variables for predicting prolonged air leak following pulmonary resection. J Thorac Dis. 2019;11:S1891–3. 31632777 10.21037/jtd.2019.08.58 PMC6783740

[5] Brunelli A, Cassivi SD, Halgren L. Risk factors for prolonged air leak after pulmonary resection. Thorac Surg Clin. 2010;20:359–64. 20619226 10.1016/j.thorsurg.2010.03.002

[6] Rivera C, Bernard A, Falcoz PE, Thomas P, Schmidt A, Bénard S, et al. Characterization and prediction of prolonged air leak after pulmonary resection: a nationwide study setting up the index of prolonged air leak. Ann Thorac Surg. 2011;92:1062–8. 21871301 10.1016/j.athoracsur.2011.04.033

[7] Gilbert S, Maghera S, Seely AJ, Maziak DE, Shamji FM, Sundaresan SR, et al. Identifying patients at higher risk of prolonged air leak after lung resection. Ann Thorac Surg. 2016;102:1674–9. 27457828 10.1016/j.athoracsur.2016.05.035

[8] Attaar A, Winger DG, Luketich JD, Schuchert MJ, Sarkaria IS, Christie NA, et al. A clinical prediction model for prolonged air leak after pulmonary resection. J Thorac Cardiovasc Surg. 2017;153:690-699.e2. 27912898 10.1016/j.jtcvs.2016.10.003 PMC5651171

[9] Pompili C, Falcoz PE, Salati M, Szanto Z, Brunelli A. A risk score to predict the incidence of prolonged air leak after video-assisted thoracoscopic lobectomy: an analysis from the European Society of Thoracic Surgeons database. J Thorac Cardiovasc Surg. 2017;153:957–65. 28089646 10.1016/j.jtcvs.2016.11.064

[10] Elsayed H, McShane J, Shackcloth M. Air leaks following pulmonary resection for lung cancer: is it a patient or surgeon related problem? Ann R Coll Surg Engl. 2012;94:422–7. 22943333 10.1308/003588412X13171221592258 PMC3954324

[11] Okada S, Shimada J, Kato D, Tsunezuka H, Inoue M. Prolonged air leak following lobectomy can be predicted in lung cancer patients. Surg Today. 2017;47:973–9. 28091813 10.1007/s00595-016-1467-5

[12] Petrella F, Rizzo S, Radice D, Borri A, Galetta D, Gasparri R, et al. Predicting prolonged air leak after standard pulmonary lobectomy: computed tomography assessment and risk factors stratification. Surgeon. 2011;9:72–7. 21342670 10.1016/j.surge.2010.07.010

[13] Zhao K, Mei J, Xia C, Hu B, Li H, Li W, et al. Prolonged air leak after video-assisted thoracic surgery lung cancer resection: risk factors and its effect on postoperative clinical recovery. J Thorac Dis. 2017;9:1219–25. 28616271 10.21037/jtd.2017.04.31 PMC5465145

[14] Tong BC, Kosinski AS, Burfeind WR, Onaitis MW, Berry MF, Harpole DH, et al. Sex differences in early outcomes after lung cancer resection: analysis of the Society of Thoracic Surgeons General Thoracic Database. J Thorac Cardiovasc Surg. 2014;148:13–8. 24726742 10.1016/j.jtcvs.2014.03.012 PMC4314218

[15] Bluman LG, Mosca L, Newman N, Simon DG. Preoperative smoking habits and postoperative pulmonary complications. Chest. 1998;113:883–9. 9554620 10.1378/chest.113.4.883

[16] Dales RE, Dionne G, Leech JA, Lunau M, Schweitzer I. Preoperative prediction of pulmonary complications following thoracic surgery. Chest. 1993;104:155–9. 8325061 10.1378/chest.104.1.155

[17] Nakagawa M, Tanaka H, Tsukuma H, Kishi Y. Relationship between the duration of the preoperative smoke-free period and the incidence of postoperative pulmonary complications after pulmonary surgery. Chest. 2001;120:705–10. 11555496 10.1378/chest.120.3.705

